# The Psychological State of Teachers During the COVID-19 Crisis: The Challenge of Returning to Face-to-Face Teaching

**DOI:** 10.3389/fpsyg.2020.620718

**Published:** 2021-01-12

**Authors:** Naiara Ozamiz-Etxebarria, Naiara Berasategi Santxo, Nahia Idoiaga Mondragon, María Dosil Santamaría

**Affiliations:** ^1^Department of Developmental and Educational Psychology, University of the Basque Country, Leioa, Spain; ^2^Department of Didactics and School Organization, University of the Basque Country, Leioa, Spain; ^3^Department of Research and Diagnostic Methods in Education, University of the Basque Country, Leioa, Spain

**Keywords:** education, teachers, stress, anxiety, depression, COVID-19

## Abstract

Schools in Spain were closed in March 2020 to prevent the spread of COVID-19. In September 2020 most schools and universities in Spain reopened and teachers felt great uncertainty due to this unprecedented situation. Teachers have accumulated psychological symptoms since the beginning of the pandemic. During the lockdown they had to introduce online teaching and in view of the reopening of schools they have shown great concern for the new unprecedented teaching situation. The present study aims to measure the symptomatology shown by teaching staff in the Basque Autonomous Community at the time when schools were reopened. To do this, we recruited a sample of 1,633 teachers who were given an online questionnaire which, in addition to collecting socio-demographic data, measured stress, anxiety and depression using the DASS-21scale. The results revealed that a high percentage of teachers showed anxiety, depression and stress symptoms. Furthermore, variables such as gender, age, job stability, the level of education at which they teach and parental status also influence this symptomatology. We argue for the need to safeguard the mental health of teachers in order to improve both the quality of teaching and the mental health of students.

## Introduction

The COVID-19 pandemic has changed our understanding of the world as we previously knew it. The strong measures of social distancing and lockdown that have been applied since the beginning of the pandemic have led to significant changes in social relationships, which, for many people, have created feelings of isolation and loneliness (Smith and Lim, 2020).

Indeed, the rapid transmission of COVID-19 throughout the world has had health, social, psychological, economic and, of course, educational consequences. In fact, school closure has been, on a global scale, one of the most widely used measures for helping to maintain social distancing and to decrease infections (Sheikh et al., 2020; Van Lancker and Parolin, 2020; Viner et al., 2020).

In fact, the United Nations Educational, Scientific and Cultural Organization (UNESCO), 2020b estimated that at the end of March 2020, 1.574,989,812 students (90% of those enrolled) in the world were affected by the closure of schools due to COVID-19. Eight months later, with the beginning of the 2020–2021 school year, there were still 851,870,246 students in the world affected by school closures (48.7% of those enrolled).

In the Basque Country, a territory in the north of Spain, the first restrictive measure that was implemented in response to the COVID-19 pandemic was the closure of all education centers. And on March 12, 2020, the Basque Government closed all schools and universities (Basque Government Health Department, 2020). Two days later, on March 14, the Spanish Government declared a state of emergency, extending this restriction to the whole country (Gobierno de España, 2020b). From that moment, a strict lockdown began, and Spanish children and adolescents were confined to their homes for more than 6 weeks (Idoiaga et al., 2020a,b), after which a de-escalation phase began in which the restrictions were gradually eased until the beginning of July (Gobierno de España, 2020a). Therefore, school and university teaching were transformed overnight into a virtual modality and remained this way throughout the 2019–2020 academic year (Al Lily et al., 2020; Besser et al., 2020; Kim and Asbury, 2020).

### The Response of Teachers to the Pandemic

The pandemic has not only affected the mental state of students (Cachón-Zagalaz et al., 2020), since teachers have also accumulated a high level of stress since the beginning of the crisis. Recent studies have pointed out that during lockdown, teachers have suffered stress from having to adapt (in record time) in order to provide online classes (Besser et al., 2020). This stress has often been accompanied by symptoms of anxiety, depression, and sleep disturbance as a consecuence of the increased workload resulting from home teachi Ng (2007).

Not many studies conducted during the pandemic measure the symptoms of stress, anxiety, and depression among teachers but the studies that have been carried out suggest that they have psychological symptoms and this reinforces the importance of reopening schools and universities. A recent Arab study has indicated that this crisis has caused teachers to suffer problems that are often related to a pandemic situation, such as anxiety, depression, domestic violence, and divorce, all of which restrict their ability to teach properly (Al Lily et al., 2020). A study carried out in three cities in China during the pandemic assessed the prevalence of anxiety among teachers and found a prevalence of 13.67%, with women being more anxious than men and the older ones being more symptomatic (Li et al., 2020). Another study conducted in March also in China showed that the prevalence of stress symptoms in teachers was 9.1% and that it was important to support them psychologically (Zhou and Yao, 2020). In a study conducted in Spain at the beginning of the panemic, teachers also reported having workloads, psychosomatic problems, and exhaustion (Prado-Gascó et al., 2020).

Moreover, previous studies have found that working from home using Information and Communication Technologies (ICT) can create feelings of tension, anxiety, exhaustion, and decreased job satisfaction (Cuervo et al., 2018), and in times of a pandemic these were the only tools that were available to teachers.

United Nations Educational, Scientific and Cultural Organization (UNESCO) (2020a) has already identified confusion and stress among teachers as being one of the adverse consequences of school closures, due to the abruptness of such measures, uncertainty about their duration, and a lack of familiarity with distance education. The unpleasant work-related emotions associated with the depletion of psychological resources has long been a topic of frequent discussion among education professionals, policy makers, and researchers (Kim and Asbury, 2020). This may occur because the long-term nature of the problem leads to exhaustion by creating less confidence in their ability to do their jobs and makes it more difficult to manage student behavior (Burić and Kim, 2020).

To this new context, it should be added that the teaching profession has always brought with it added stress due to excessive workloads, interpersonal communication problems, insufficient training, and job insecurity (Pérez, 2003). Research carried out in several countries has revealed that in the teaching profession there have been many casualties of stress, anxiety, and depression (Ryan et al., 2017; Von der Embse et al., 2019). In fact, psychological symptomatology has been studied in both primary (Extremera et al., 2010; Skaalvik and Skaalvik, 2016; Abdullah and Ismail, 2019) and secondary (Betoret, 2006, 2009) educators as well as in university teachers (Malik et al., 2017; Puertas-Molero et al., 2018). Although more psychological symptomatology has been detected in secondary school educators (Arias et al., 2019), in comparison with those working in primary schools, other variables such as salary, relationships with students, and relationships with classmates are also important factors (Prieto and Bermejo, 2006). This stress may have consequences for the health of teachers and, as a result, coud lead to increased instances of sick leave, absenteeism, and poor work performance (Moreno et al., 2004). Further, it is important to safeguard the emotional health of teachers since, as a recent study by De la Fuente et al. (2020) highlights, teacher–student relationships are also stressors for the student, and the teacher’s behavior predicts the emotional well-being and commitment of the students, which are also important factors for reducing their stress levels.

Another issue worthy of consideration is the fact that the COVID-19 pandemic has not only created a health crisis but also an extremely significant global economic downturn, the effects of which have been particularly harsh in Spain (Torres and Fernández, 2020). In fact, the job instability of teachers was an issue that had already attracted attention before the pandemic (García and Martín, 2012) and COVID-19 has only served to exacerbate this problem with more layoffs and instability (Aunión and Romero, 2020; La Vanguardia., 2020). Several investigations analyzing the impact of job instability on teachers have shown that this can have significant psychological consequences (Leibovich, and de Figueroa, 2006).

Amid this context of uncertainty, the 2020–2021 academic year has approached without any clear decision on how it will be played out (Zafra, 2020). In fact, since the end of August, families, students, teachers and educational centers have been expressing their concerns about the uncertainty surrounding the new academic year and the lack of clear guidelines from the government (Rioja, 2020). On August 27, the Spanish Government and the Autonomous Communities at the Education Sector Conference agreed on the main measures that would be adopted for a return to the classroom (Sanchez, 2020). On August 28, the Basque government’s civil protection monitoring commission reported on the measures to be taken in the new 2020–2021 year (Basque Government Health Department, 2020). However, the way in which these measures could be implemented with the resources available to the schools emerged as a considerable challenge and source of concern only one week before the beginning of the school year on September 7 (Lucas, 2020).

Whilst classes reopened in September, teachers and education centers were not satisfied with the guidelines that they were required to follow. Moreover, responsibility for the health measures was in the hands of each individual school, high school, and university (Zafra, 2020). As a consequence of this situation, and in view of the sense of unease that it created, a strike of non-university teachers was called and carried out on September 15 in the Autonomous Community of the Basque Country, with approximately 50–70% of teachers participating in this action (Navarro, 2020).

### Aims and Hypothesis

Work conducted within academia suggests that the immediate priority of the research community during this unprecedented time should be to reduce mental health problems and support the well-being of vulnerable groups. The COVID-19 pandemic could have a long-lasting impact on teachers and teaching activities and, as a consequence, on children and adolescents (Holmes et al., 2020).

By considering how teachers are coping with the return to school during this pandemic we might be in a better position to put in place the relevant support structures that may be needed (Dalton et al., 2020; Holmes et al., 2020; Wang et al., 2020). Therefore, the impact of the COVID-19 pandemic on the emotional well-being of teachers is a major challenge that needs to be tackled by both the educational community and society in general. Although amid this pandemic we have already witnessed rising levels of symptomatology in society as a whole and in members of certain professions such as the healthcare sector (Chen et al., 2020; Dosil et al., 2020), this reality has not yet been studied among teaching staff in Spain. Thus, the main objective of this study is to evaluate the emotional state of teachers at a critical moment in the current crisis, that is, during the reopening of schools and educational centers of the Basque Autonomous Community following a six-month hiatus to prevent the transmission of COVID-19.

In particular, we aimed to analyze the levels of depression, anxiety, and stress that have been experienced by teachers at the beginning of the 2020–2021 school year and how this symptomatology was affected by the sector in which they work (pre-school, primary, secondary, vocational, or university education) along with their job stability. Differences in symptoms will also be analyzed according to gender, age and whether they had school-age children who were also facing a return to school. In addition, it was also analyzed if being infected with COVID-19 or lockdowned at the time of answering the questionnaire or if someone close to them had been sick with COVID-19 affected the teachers’ answers.

We expected to observe high levels of symptomatology, with women, younger people, and those with children being most affected. It was also anticipated that the people with the greatest instability in the workplace will be those who display the most symptoms, and that secondary school teachers will be the ones who experience the greatest degree of psychological discomfort.

## Materials and Methods

### Participants

This study was carried out with a total sample of 1,633 teachers from compulsory and non-compulsory education (from nursery education to university studies) from the various education centers (public and private) of the Basque Autonomous Community and Navarre (Spain). Of the sample, 79.7% were women (*n* = 1293), and 20.3% were men (*n* = 330), with an average age of 42.6 years (SD = 9.96). In terms of sector, 18.9% were teaching in Pre-school Education (*n* = 309), 32.55% in Primary Education (*n* = 530), 30.1% in Secondary Education (*n* = 491), 5.5% Bachelor Studies (*n* = 89), 5.6% Vocational Training (*n* = 91), and 7.5% were teaching University Studies (*n* = 123).

### Measures and Instruments

A questionnaire was designed to gather socio-demographic data (gender and age), whether they were infected with COVID-19 or lockdowned whether someone close to them had been sick with COVID-19, information on parental status, and questions about the duration of their employment contract along with the sector in which they are teaching.

The Spanish version of the Depression Anxiety and Stress Scale-21 [DASS-21, Ruiz et al., 2017] was employed. This instrument consists of 21 items with 4 response options (from 0 = did not occur to 3 = occurred a lot or most of the time) that are grouped into three factors: depression, anxiety, and stress: Depression (items: 3, 5, 10, 13, 16, 17, and 21), Anxiety (items: 2, 4, 7, 9, 15, 19, and 20), and Stress (items: 1, 6, 8, 11, 12, 14, and 18). For the study, cut-off points were used: no symptoms, mild, moderate, severe and extremely severe symptoms. Regarding the reliability of the scale, the Cronbach’s alpha coefficient was α = 0.76 for the depression scale, α = 0.82 for the anxiety scale, and α = 0.75 for the stress scale.

### Procedure

The study was approved by the Ethics Committee of the UPV/EHU (code M10/2020/070). The initial contact was made through e-mail and the answers were collected through an online questionnaire between September 5 and 28, 2020 (beginning of the school year). A total of 1658 people participated in the study; however, 25 questionnaires had to be withdrawn because they had more than 50% of the answers blank. For the collection of the data, all the requirements established by the Organic Law 15/99 of Personal Data Protection were followed, that is, all the participants gave their approval before the beginning of the questionnaire.

Likewise, before beginning the questionnaire that measures the different symptoms (previously used in a Spanish sample because of its reliability in the joint analysis of the three symptoms) the participants have at their disposal a series of instructions from the questionnaire (description of the sample, specifically the Likert type responses of the questionnaire, as well as its objective).

### Data Analysis

The data were analyzed using the statistical program IBM SPSS Statistics for Windows, Version 26.0 (Armonk, NY, United States). Two assumptions of normality and homogeneity of variations were then demonstrated prior to conducting the corresponding analysis to decide whether parametric or non-parametric tests were used. Specifically, the critical level of *p* < 0.05 of the Kolmogorov–Smirnov statistic was analyzed to determine the distribution of the data for the analysis of group differences.

Depression, anxiety and stress were categorized using the cut-off scores of the instrument to establish the different levels (mild, moderate, severe, and extremely severe). First, both the frequencies and the percentages of the socio-demographic variables were described, after which a series of tests were carried out. On the one hand, *t-*student was carried out for variables with two groups and an ANOVA was carried out in order to observe the differences of the dependent variables in the case of having an independent variable in three groups (age, work stability, and teaching subject). For the difference between the groups, Bonferroni’s tests between groups were used.

## Results

Table 1 shows the prevalence data for teachers suffering from depression, anxiety and stress.

**TABLE 1:** Frequency and percentages of teachers that suffer from depression, anxiety, and stress.

		*n* (%)
**Depression**	None	1107(67.8)
	Mild	208(12.7)
	Moderate	195(11.9)
	Severe	70(4.3)
	Extremely severe	53(3.2)
**Anxiety**	None	825(50.5)
	Mild	200(12.2)
	Moderate	351(21.5)
	Severe	124(7.6)
	Extremely severe	133(8.1)
**Stress**	None	806(49.4)
	Mild	263(16.1)
	Moderate	260(15.9)
	Severe	230(14.1)
	Extremely severe	74(4.5)

Of the sample, 50.6% of the teachers indicated that they were suffering from stress, with 4.5% reporting extremely severe stress and 14.1% severe stress. About 49.5% of the teachers reported suffering from anxiety, 8.1% of which reported extremely severe symptoms and 7.6% severe symptoms. Finally, 32.2% of the teachers reported suffering from depression, of which 3.2% reported extremely severe symptoms and 4.3% severe symptoms.

Comparison tests were conducted using *t*-student and ANOVAs to explore whether gender, age, being infected or locked up, infection of close relatives, parental status, length of contract, and education sector were related to levels of depression, anxiety, and stress Of all these variables the ones that were not significant were to be infected with COVID-19 or lockdowned and to have a close relative who had been sick with COVID-19. The following Tables 2–4 show the descriptive statistics of the significant variables and relations that were statistically significant.

**TABLE 2:** Means, standard deviations and group differences for depression according to gender, age, parental status, work stability, and teaching sector.

	Depression						
**Gender**	***M***	***SD***	***n***	***t***	***df***	***p***	
Female	3.77	4.04	1293	1.34	1629	0.660	
Male	3.66	4.02	330				
**Age**	***M***	***SD***	***n***	***F***	***df***	***p***	***Post hoc***
23–35years (1)	3.52	3.97	451	1.63	2	0.196	
36–46 years (2)	3.71	3.84	578				
47 years or older (3)	7.52	4.58	451				
**Children**	***M***	***SD***	***n***	***t***	***df***	***p***	
Yes	3.66	4.04	772	0.049	1631	0.825	
No	3.83	4.02	861				
**Work stability**	***M***	***SD***	***n***	***F***	***df***	***p***	***Post hoc***
Less than 3 months (1)	5.42	5.91	54	3.53	3	0.014**	1 > 2,3,4
Between three months and one year (2)	3.33	3.75	102				
One year and more (3)	3.67	3.87	503				
Indef (4)	3.74	3.99	974				
**Teaching subject**	***M***	***SD***	***n***	***F***	***df***	***p***	***Post hoc***
Pre-school Education (1)	3.62	4.04	309	0.350	5	0.882	
Primary Education (2)	3.68	3.89	530				
Secondary Education (3)	3.81	4.01	491				
Bachelor (4)	3.61	4.07	89				
University studies (5)	3.93	3.97	123				
Vocational training (6)	4.14	4.90	91				

**p < 0.01.

The data in Tables 3, 4 indicate that gender differences emerged with regard to anxiety [*t* (1,621) = 4.65, *p* > 0.001] and stress [*t* (1,621) = 3.94, *p* > 0.001] with women showing higher scores on anxiety (*M* = 4.28) and stress (*M* = 8.09) versus men’s anxiety (*M* = 3.26) and stress (*M* = 6.96). With regard to age, differences were found for anxiety [*F* (2, 1,629) = 5.50, *p* = 0.004, η^2^ = 0.117] and stress [*F* (2, 1,629) = 4.77, *p* = 0.009, η^2^ = 0.109]. In particular, older participants (>47 years) showed greater levels of anxiety (*M* = 8.35) and stress (*M* = 7.69), whilst the younger participants (23–35 years) showed higher stress scores (*M* = 7.52) than those aged between 36 and 46 years.

**TABLE 3:** Means, standard deviations and group differences for anxiety according to gender, age, parental status, work stability, and teaching sector.

	Anxiety						
**Gender**	***M***	***SD***	***n***	***t***	***df***	***p***	
Female	4.28	3.59	1293	3.92	1629	0.001***	
Male	3.26	3.31	330				
**Age**	***M***	***SD***	***n***	***F***	***df***	***p***	***Post hoc***
23–35years (1)	4.29	3.78	451	5.50	2	0.004**	1 < 2,3, 2 < 3,
36–46 years (2)	4.33	3.38	578				
47 years or older (3)	8.35	4.62	578				
**Children**	***M***	***SD***	***n***	***t***	***df***	***p***	
Yes	4.05	3.60	772	0.049	1631	0.825	
No	4.13	3.55	861				
**Work stability**	***M***	***SD***	***n***	***F***	***df***	***p***	***Post hoc***
Less than 3 months (1)	5.48	4.45	5.48	3.43	3	0.016***	1 > 2,3,4
Between three months and one year (2)	3.76	3.24	102				
One year and more (3)	4.20	3.73	503				
Indef (4)	3.99	3.45	974				
**Teaching subject**	***M***	***SD***	***n***	***F***	***df***	***p***	***Post hoc***
Pre-school Education (1)	4.35	3.72	309	4.55	5	0.001***	1 > 5 2 > 5
Primary Education (2)	4.45	3.58	530				
Secondary Education (3)	3.95	3.50	491				
Bachelor (4)	3.95	3.50	491				
University studies (5)	2.95	3.00	123				
Vocational training (6)	3.86	4.01	91				

*p < 0.05; **p < 0.01 and ***p < 0.001.

**TABLE 4:** Means, standard deviations and group differences for stress according to gender, age, parental status, work stability, and teaching sector.

	Stress						
**Gender**	***M***	**SD**	***n***	***t***	***df***	***p***	
Female	8.09	4.58	1293	3.28	1629	0.001***	
Male	6.96	4.89	330				
**Age**	***M***	SD	***n***	***F***	***df***	***p***	***Post hoc***
23–35years (1)	7.69	4.58	451	4.77	2	0.009**	1 > 2,3 2 < 3
36–46 years (2)	3.71	3.57	603				
47 years or older(3)	7.52	4.75	603				
**Children**	***M***	SD	***n***	***t***	***df***	***p***	
Yes	8.10	4.80	772	3.99	1631	0.046*	
No	7.67	4.54	861				
**Work stability**	***M***	SD	***n***	***F***	***df***	***p***	***Post hoc***
Less than 3 months (1)	8.64	5.52	54	2.64	3	0.048*	1 > 2,3,4
Between three months and one year (2)	6.88	4.49	102				
One year and more (3)	7.69	4.79	503				
Indef (4)	8.03	4.56	974				
**Teaching subject**	***M***	SD	***n***	***F***	***df***	***p***	***Post hoc***
Pre-school Education (1)	7.77	4.73	309	5.24	5	0.943	
Primary Education (2)	7.91	4.42	530				
Secondary Education (3)	7.91	4.42	530				
Bachelor (4)	7.48	4.74	89				
University studies (5)	8.04	4.57	123				
Vocational training (6)	7.73	5.48	91				

*p < 0.05; **p < 0.01 and ***p < 0.001.

Differences also emerged according to whether or not the participants have school-aged children, with those having school-aged children showing higher levels of stress [*F* (3, 1,612) = 2.68, *p* = 0.046, η^2^ = 0.081], although the effect size was small.

Finally, with regard to employment stability, those with an employment contract of less than 3 months showed the highest scores on depression [*F* (3, 1,629) = 23.53, *p* = 0.014, η^2^ = 0.241], anxiety (*F* (3, 1629) = 3.43, *p* = 0.016, η^2^ = 0.092] and stress [*F* (3, 1,629) = 2.64, *p* = 0.048, η^2^ = 0.081]. Likewise, differences emerged with regard to teaching sector [*F* (5, 1,627) = 4.55, *p* = 0.001, η^2^ = 0.106] with primary and secondary school teachers showing higher scores on anxiety compared with those teaching in the university sector.

## Discussion

The results of this study have confirmed that a high percentage of teachers suffered from symptoms of anxiety, stress and depression when the schools and universities reopened. These symptomatology rates are somewhat high in comparison with those reported in another study conducted during the pandemic with the general population in the same area of Spain (Ozamiz-Etxebarria et al., 2020). Nonetheless, these findings are in line with those of other studies carried out from the beginning of the pandemic showing that during lockdown, teachers have suffered from stress (Besser et al., 2020), anxiety (Huang and Zhao, 2020), and other psychological and physical symptoms (United Nations Educational, Scientific and Cultural Organization (UNESCO), 2020b; Aperribai et al., 2020). However, this study shows that this symptomatology is not only characteristic of periods of lockdown. In fact, in this study no significant differences were found between teachers who were confined and those who were not.

Among the psychological symptoms evaluated here, the most striking findings to emerge are those related to stress. Whilst it is true that according to some studies, stress levels were already high among teachers before the onset of the pandemic (Hadi et al., 2009; Teles et al., 2020), the results of this study reveal that the levels of symptomatology are considerably higher than those the teachers could have suffered prior to the pandemic, showing that more than half of them have suffered from stress, with 18.6% of these cases being severe or extremely severe. These high rates of symptomatology among teachers could be due to the academic context in which they work and the new measures they have had to adopt without the necessary support in the form of material and human resources (Navarro, 2020). However, it should be borne in mind that this level of stress could also be linked to uncertainty about the possibility of children becoming infected in schools. In other words, since the beginning of the pandemic it has been suggested that schools could be a major source of the disease (Soriano, 2020), and although recent studies suggest otherwise (Zafra, 2020), the possibility of schools becoming a focus of the disease could also contribute toward the increase in stress levels. Therefore, returning to the classroom beyond being a return to normality seems to have become a new focus of uncertainty for teachers.

Also in this study several factors have been found that directly affect that symptomatology. Some of them are personal and others professional. Starting with the personal factors in terms of gender, this study indicates that there are higher levels of stress and anxiety among women in comparison with men. Pre-pandemic studies in the general population have already indicated that women are at significantly greater risk of suffering from symptoms of anxiety and stress (Arenas and Puigcerver, 2009; Soffer, 2010). During the pandemic, studies conducted with health professionals (Dosil et al., 2020; Huang et al., 2020; Rajkumar, 2020) and the general population (Liu et al., 2020) have continued to show this trend of greater symptomatology in women than in men. This factor, although personal, is also characteristic of the profession since most of the teachers in Spain are women; in fact 79.7% of the participants in this study were women. Therefore, it should be reflected whether in such a feminized profession might be especially susceptible to the pandemic. Some studies already point out that the role of caregiver (both professionally and personally) could also increase this symptomatology detected here (Dosil et al., 2020).

In a similar vein, the results show that having school-aged children also creates a greater impact on the feelings of stress. Having to deal with a heavy workload, combined with the stress of carrying out family care duties, may be one of the reasons why teachers with children have suffered from higher levels of stress. It should also be noted that these professionals had to face a double return to school – both their own and that of their children. Indeed, having a family may have played a role in the increased stress experienced during the pandemic (Fitzpatrick et al., 2020), since managing a family in such times of crisis may bring with it different stresses, such as financial worries, or the additional burdens of parenting, childcare, or the demands of home schooling (Daks et al., 2020).

In terms of age, it is noteworthy that people over 47 years old are those who reported the highest levels of anxiety and stress, given that in other studies it is young people who have shown more symptoms (Picaza et al., 2020). This could be due to the fact that among teachers there are more symptoms of stress and anxiety that are the result of higher burnout (Corbin et al., 2019) and it could be the case that older people are currently feeling this symptomatology to a greater degree. Moreover, in this new situation that requires adaptability to all new technologies, younger people may have fewer difficulties whereas older people may be less interested in ICT (Song and Chen, 2019). This factor also goes partly beyond the personal since the aging of the teaching staff is a reality in Spain. Therefore, it has to be taken into account when designing coping strategies for the pandemic.

On the other hand the higher levels of stress among young people (23–35 years), compared with those aged 36–46 years is in line with the general trend observed in other studies, that is, younger people experience higher levels of stress (Lai et al., 2020). This may also be closely linked to job insecurity, a factor that has not been analyzed in the pandemic so far and that may be relevant due to the great adjacent economic crisis it is creating in countries like Spain. In fact, this study has demonstrated that those who have greater job instability show higher scores on depression, anxiety and stress. This variable can have very serious implications since it was demonstrated even before the pandemic that teachers with unstable contracts showed poorer health (both physical and mental) in spite of being young (Cladellas-Pros et al., 2018). Therefore increased job instability due to the pandemic will also be a factor negatively impacting the health of teachers and therefore urgent steps should be taken to stabilize contracts.

Furthermore, teachers working in the earlier stages of pre-school and primary education are those who showed the highest scores on anxiety. This marks a clear difference with previous studies in non-pandemic situation where high school teachers were the most affected by psychological symptoms (Arias et al., 2019). This could be due to the fact that (among other things) these teachers feel a greater responsibility for the younger children that need more care and protection because of their age, and they may feel very pressured to carry out these duties of care adequately by responding to the needs of the children and the concerns of their families. Moreover, stricter measures are being taken with younger students including “bubble” classes where students in one class cannot interact with other classes, whereas secondary school and university students are more autonomous and do not require as much care from teachers. Therefore, a system of educational reinforcement to be able to carry out all the tasks that are demanded of them would be convenient to reduce this psychological discomfort.

Until now, no studies in Spain have analyzed the psychological impact of the return to schools on teachers during a pandemic, and this is the main strength of this study. Moreover, our findings could have relevant practical implications. First, it would be highly desirable to decrease the psychological impact on teaching professionals (particularly women, older teachers, those with less job security, and those who have children in their care). To this end, it would be beneficial to provide psychological support through telephone or face-to-face assistance. Further, it would be helpful to create more optimal communication channels for gathering information about COVID-19 and its implications for schools. In fact, the lack of clarity with regard to appropriate guidelines along with the scarce resources available has posed a great challenge and is a problem that needs to be urgently addressed within the entire school community (Lucas, 2020).

Regarding the limitations of the study, the generalizability of our results is limited, since we used a non-probabilistic sample. In particular, there may be a certain selection bias, since participation was voluntary, and therefore only those who were particularly emotionally affected could have participated. Furthermore, the cross-sectional nature of the study does not allow a comparison between pre-pandemic and post-pandemic results. Moreover, variables related to the impact of the pandemic have not been taken into account such as whether people close to them had died from COVID-19 or whether they lived in areas with a particular constraint. Future studies should use a more extensive sample, including participants from more autonomous communities. They should also take into account more variables related to the direct impact of the pandemic.

## Conclusion

Members of the teaching profession experienced psychological discomfort at the beginning of the new 2020–2021 academic year. This symptomatology has been found to be higher in women than in men, but, contrary to our expectations, was found be higher in older people and in teachers of infant and primary education. As expected, people with job instability are those who have suffered the most psychological symptoms. These findings indicate the importance of safeguarding the mental health of teachers to ensure both the well-being of students and high quality teaching. Therefore, special psychological care should be given to those teachers that are most vulnerable to the impact of this pandemic, so that they could better cope with this crisis, and consequently perform better in their teaching role. In short, we must bear in mind that the education received by young people in this current time of crisis will shape the society of the future. Therefore, if we want this education to be of a high standard, then we must protect the psychological well-being of the people who provide it.

## Data Availability Statement

The raw data supporting the conclusions of this article will be made available by the authors, without undue reservation.

## Ethics Statement

The studies involving human participants were reviewed and approved by the Ethics Committee for Research Related to Human Beings (CEISH) of the UPV/EHU. The participants provided their written informed consent to participate in this study.

## Author Contributions

NI and NO-E were involved in the conceptualization of the project and in the acquisition of the data. NB and MD were involved in the analysis and interpretation of the data. All authors were involved in the drafting and revising of the work for intellectual content, provided approval for submission of the contents for publication, and agreed to be accountable for the accuracy and integrity of the project.

## Conflict of Interest

The authors declare that the research was conducted in the absence of any commercial or financial relationships that could be construed as a potential conflict of interest.
